# A new species of *Talaromyces* sect. *Subinflati* discovered in China

**DOI:** 10.7717/peerj.21395

**Published:** 2026-06-01

**Authors:** Wei Zang, Junxia Yin, Siyuan He, Jianqiu Sun, Long Wang

**Affiliations:** 1 School of Life and Environmental Science, Shaoxing University, Shaoxing, Zhejiang, China; 2 Mycology and Innovative Technology Laboratory, Institute of Microbiology, Chinese Academy of Sciences, Beijing, China

**Keywords:** Penicillia, Polyphasic taxonomy, Saprotrophic fungi, Soil fungi

## Abstract

A new species of *Talaromyces* sect.* Subinflati* isolated from soil in China is proposed based on polyphasic taxonomy, namely, *T. bawangicus*. Morphologically, this new taxon is characterized by limited growth on culturing media Czapek yeast autolysate agar (CYA), yeast extract sucrose agar (YES), Czapek solution agar (Cz) and oatmeal agar (OA), with sparse sporulation on CYA, Cz, OA, while abundant sporulation on YES, and moderate growth and sporulation on 5% malt extract agar (MEA), producing apically vesiculate stipes, loose biverticillate penicilli with metulae and phialides unequal in length, bearing ampuliform phialides and smooth-walled, ovoid, ellipsoidal to fusiform conidia. The proposed taxonomic novelty is supported by the phylogenetic analysis of the concatenated *BenA-CaM-Rpb2-*ITS sequence matrix. The new species is in the clade including *Talaromyces guizhouensis*, *Talaromyces jiangxiensis*, *Talaromyces sinensis*, *Talaromyces subinflatus* and *Talaromyces tzapotlensis*, closely related to *T. jiangxiensis*.

## Introduction

*Talaromyces* species (Eurotiomycetes, Trichocomaceae) are widely distributed in terrestrial, aquatic (freshwater and marine) and atmospheric environments, and often found in association with materials of plants, animals, humans, or even fungi (*e.g.*, Luo et al., 2016; Chen et al., 2016; Wang et al., 2016; Guevara-Suarez et al., 2017; Barbosa et al., 2018; Rodríguez-Andrade et al., 2019; Wang et al., 2020; Trovão et al., 2021; Han, Sun & Wang, 2022; Sun et al., 2022). As the common saprobes, *Talaromyces* species play an important ecological role in the nutrient cycle in natural ecosystems, producing various lignocellulolytic enzymes engaging in plant biomass degradation, especially *Talaromyces pinophilus* and *Talaromyces funiculosus* (*e.g.*, Pasari et al., 2023; Fujii, 2025; Nie et al., 2025). In addition to the vegetal biomass degradation, *Talaromyces* species secret organic acids and phosphate-solubilizing enzymes to demineralize phosphate in soil, actively participating in the phosphorus cycle in nature (Ölmez et al., 2024; Sun et al., 2025). Besides the saprobic role, many *Talaromyces* species are endophytes in various plant species and families, which are beneficial to plant health, such as promoting growth or antagonizing pathogens; moreover, these endophytes biosynthesize various bioactive secondary metabolites, some as certain active ingredients of the medicinal hosts (Nicoletti, Andolfi & Salvatore, 2023). Additionally, *Talaromyces* species have also been found associated with insects, such as inhabiting the galls of cynipid wasps, anthills of leaf-cutting ants, combs of wasps, and nests of termites, or even the gut of certain insects (Nicoletti & Becchimanzi, 2022). However, some *Talaromyces* species are opportunistic pathogens causing invasive talaromycosis in immunosuppressed or immunocompromised patients. For instance, talaromycosis caused by *Talaromyces marneffei* is endemic in Southeast Asia and Southern China (*e.g.*, Zhou et al., 2025). Other *Talaromyces* species reported as pathogens are sporatic, for example, Ying et al. (2024) reported a case of pulmonary infection caused by *Talaromyces amestolkiae*, and Won et al. (2025) reported a case of fungemia by *Talaromyces tumuli*. *Talaromyces* species have a close relationship with human activities and are valuable fungal resources to be exploited, thus the reliable indentification and taxonomy of species is greatly needed.

The genus *Talaromyces* was further divided into eight sections based on the molecular phylogenetic method, and over 240 species have been reported in this genus (Houbraken et al., 2020; Visagie et al., 2024; Peng et al., 2025). As the smallest section of *Talaromyces*, *Subinflati* originally included only two members in the monograph of Yilmaz et al. (2014), namely, *Talaromyces palmae* and *Talaromyes subinflatus*. Then *Peterson and Jurjević* (2017) added a new member, *i.e.,* *Talaromyes tzapotlensis* from Mexico, and Sun et al. (2020) transferred a monoverticillate *Penicillium* species, *i.e*., *Penicillium resedanum* to this section as *Talaromyes resedanus* and added a new member, namely, *Talaromyes guizhouensis*. Later on, four new members were discovered in China, namely, *Talaromyes jiangxiensis* and *Talaromyes paecilomycetoides* (Zhang et al., 2023), *Talaromyes parapalmae* (Zhang et al., 2024), and *Talaromyes sinensis* (Peng et al., 2025). Thus, only nine species belonging to this section have been described until now (Table 1).

**Table 1:** Seventeen species, strains and GenBank accession numbers for the four genetic markers of sect. *Subinflati* and *Bacillispori* included in the molecular phylogenetic analyses.

Species	Strains	Genetic markes			
		ITS	*BenA*	*CaM*	*Rpb2*
sect. *Subinflati*					
*T. guizhouensis*	CBS 141837^T^	MN864277	MN863346	MN863323	MN863335
*T. jiangxiensis*	CGMCC 3.20783^T^	OL897029	ON569044	ON568888	ON568963
*T. paecilomycetoides*	CGMCC 3.20785^T^	OL897033	ON569040	ON568890	ON568959
*T. palmae*	CBS 442.88^T^ = IMI 343640^T^	JN899396	HQ156947	KJ885291	KM023300
*T. parapalmae*	CGMCC 3.25510^T^	OR680520	OR843225	OR828456	OR842937
*T. resedanus*	CBS 181.71^T^ = FRR 578^T^	MN431413	MN969436	MN969355	MN969214
*T. sinensis*	CGMCC 3.28744^T^	PV085755	PV102705	PV102718	PV102726
*T. subinflatus*	CBS 652.95^T^	JN899397	MK450890	KJ885280	KM023308
*T. tzapotlensis*	NRRL 35203^T^	KX946902	KX946884	KX946893	KX946922
***T. bawangicus***	**CGMCC 3.29770** ^**T**^	**PX983013**	**PX995142**	**PX995144**	**PX995146**
	**CGMCC 3.29771**	**PX983014**	**PX995143**	**PX995145**	**PX995147**
sect. *Bacillispori*					
*T. bacillisporus*	CBS 296.48^T^ = NRRL 1025^T^	KM066182	AY753368	KJ885262	JF417425
*T. clematidis*	CBS 149228^T^	ON863768	ON873763	ON938196	ON938200
*T. columbiensis*	CBS 113151^T^ = DTO 058F3^T^	KX011503	KX011488	KX011499	MN969187
*T. emodensis*	CBS 100536^T^ = IBT 14990^T^	MH862707	KJ865724	KJ885269	JF417445
*T. mimosinus*	CBS 659.80^T^ = FRR 1875^T^	JN899338	KJ865726	KJ885272	MN969149
*T. proteolyticus*	CBS 303.67^T^ = NRRL 3378^T^	JN899387	KJ865729	KJ885276	KM023301
*T. unicus*	CBS 100535^T^ = CCRC 32703^T^	JN899336	KJ865735	KJ885283	MN969150
*T. disparis* (outgroup)	AS3.26221^T^	PP544888	PP566271	PP566276	PP555175

**Notes.**

Ex-type strains are indicated with “^T^”, the new species, their strains and sequences are shown in bold face.

In a survey on *Talaromyces* species in China, we isolated two distinctive *Talaromyces* strains from two locations near the southern and southwestern land boundaries about 2,600 km apart, possibly representing a new species belonging to the section *Subinflati* represented by them, thus reported here as *Talaromyes bawangicus* sp. nov.

## Materials and Methods

### Isolation of fungal strains

Soil samples were collected from soil of the Bawang Ridge Nature Reserve, Changjiang, Hainan Province, and Chentanggou of Dingjie County, Shigaze, Tibet, China. The dilution plating method of Malloch (1981) was used for the isolation of the fungi with 0.1% agar water solution (w/v) instead of water. One ml portion of each 10^−5^ dilution and 10^−6^ dilution of the soil suspensions was plated with the Dichloran rose-bengal chloramphenicol (DRBC) medium in a 90 mm Petri dish (Pitt & Hocking, 2009), then incubated at 25 °C for 3–7 d. Two distinctive *Talaromyces* strains were obtained and deposited in the China General Microbiological Culture Collection (CGMCC) as CGMCC 3.29770 (CJ2-3) and CGMCC 3.29771 (NL1-12).

### Morphological observations

For examining colony characters, the isolates were inoculated at three points on the 90 mm Petri dishes with Czapek yeast autolysate agar (CYA), 5% malt extract agar (MEA, malt extract (Oxoid, Hants, UK), yeast extract sucrose agar (YES, yeast extract (Oxoid, Hants, UK), Czapek solution agar (Cz, BD, Sparks, USA) and oatmeal agar (OA), then incubated at 25 °C for 7 d, and the inocula on CYA were also incubated at 37 °C and 5 °C for 7 d, respectively (Samson et al., 2010; Yilmaz et al., 2014). Color names were referenced to Ridgway (1912). Microscopic traditional tease mounts were made according to Xu et al. (2022).

**Phylogenetic analysis.** For PCR amplification of the genetic markers, a tiny portion of mycelia about 0.5 mm in diameter (as the diameter of a roller pen tip) was picked up with a sterilized inoculating needle or a 10 µL pipet tip and then stuck onto the bottom part of the inner wall of the PCR tube, which was used as the amplification template. Partial *CaM*, *Rpb2* and ITS sequences were amplified with primers AD1, AD2 and Q1, Q2 (Wang, 2012); T1, T2 and E1, E2 (Jiang et al., 2018); ITS5 and ITS4 (White et al., 1990), respectively; partial *BenA* was amplified with the modified forward primer Bt2a, namely, Btga1: 5′-GGGTAACCAAATTGGTGCTGC-3′ or Btga2: 5′-GGGTAACCAAATCGGTGCTGC-3′, and the reverse primer Bt2b (Glass & Donaldson, 1995) or a modified Bt2b, *i.e*., Bt2b1: 5′-ACCTTCGGTGTAGTGACCCTTGGC-3′ to avoid amplifying *tubC*. PCR amplification was performed as previously described by Xu et al. (2022), except that the initial denatuation was set at 99 °C for 5 min. Raw sequences were proofread and edited manually with BioEdit 7.0.9 (Hall, 1999), the edited sequences without primer sequences were deposited in GenBank (CJ2-3 = CGMCC 3.29770: ITS = PX983013, *BenA* = PX995142, *CaM* = PX995144, *Rpb2* = PX995146; NL1-12 = CGMCC 3.29771: ITS = PX983014, *BenA* = PX995143, *CaM* = PX995145, *Rpb2* = PX995147).

Ten species of section *Subinflati*, including the currently identified species, and seven species of the closely related section *Bacillispori* were included in the analyses (Table 1), with *Talaromyes disparis* of section *Talaromyces* as the outgroup. The individual and the concatenated *BenA*-*CaM*-*Rpb2*-ITS sequences were aligned and analyzed as previously described by Xu et al. (2022).

### Nomenclature

The electronic version of this article in Portable Document Format (PDF) will represent a published work according to the International Code of Nomenclature for algae, fungi, and plants, and hence the new names contained in the electronic version are effectively published under that Code from the electronic edition alone. In addition, new names contained in this work have been submitted to MycoBank from where they will be made available to the Global Names Index. The unique MycoBank number can be resolved and the associated information viewed through any standard web browser by appending the MycoBank number contained in this publication to the prefix “http://www.mycobank.org/MycoTaxo.aspx?Link=T&Rec= ”. The online version of this work is archived and available from the following digital repositories: PeerJ, PubMed Central, and CLOCKSS.

## Results

### Phylogenetic analyses

**Figure 1:**
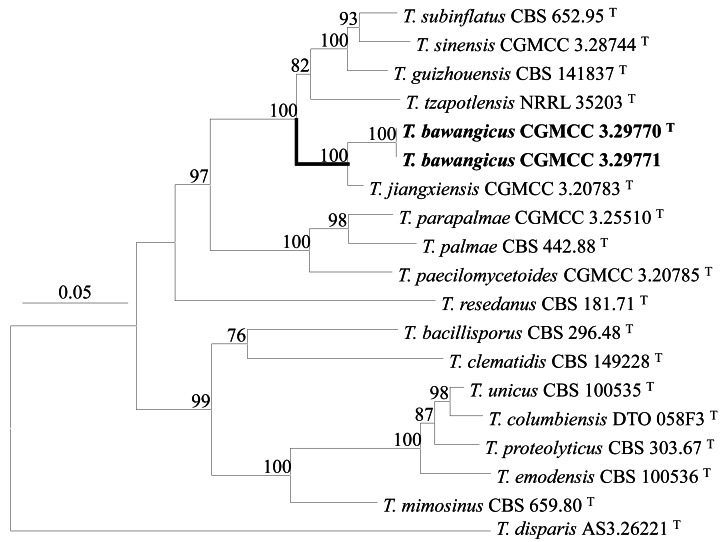
ML phylogram inferred from the concatenated BenA-CaM-Rpb2-ITS sequences. Bootstrap percentages over 70% derived from 1,000 replicates. Bar = 0.05 substitutions per nucleotide position.

PCR amplification of *BenA*, *CaM* and *Rpb2*, ITS generated 404 bp, 669 bp and 826 bp, 576 bp amplicons, respectively. The sequence matrices of *BenA*-*CaM*-*Rpb2-* ITS, *BenA*, *CaM*, *Rpb2* and ITS contain 2011, 339, 493, 678 and 465 sites with gaps, respectively. The resulted phylograms inferred from the concatenated *BenA-CaM-Rpb2*-ITS and individual sequence matrices all supports the proposal of the new species, namely, the two isolates form a distinctive clade closely related to *T. jiangxiensis* in a cluster with *T. guizhouensis*, *T. jiangxiensis*, *T. sinensis*, *T. subinflatus*, *T. tzapotlensis* (Fig. 1, Figs. S1–S4).

### Taxonomy

**Table:** 

**Figure 2:**
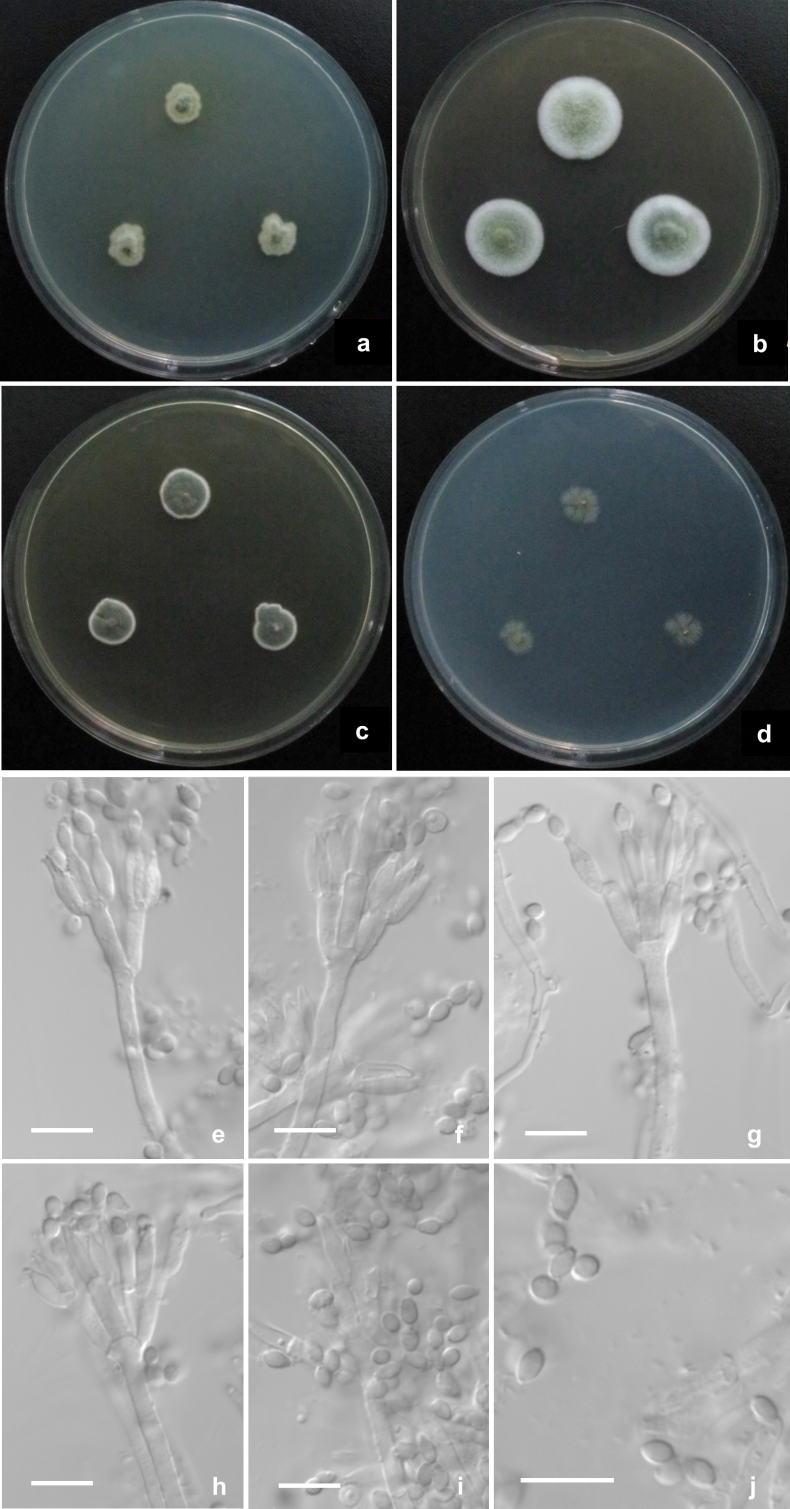
Morphological characters of *T. bawangicus* CGMCC 3.29770 T incubated at 25 °C for 7 d. (A–D) Colonies on CYA, MEA, YES and Cz. (E–H) Conidiophores. (I and J) Conidia. Scale bar = 10 µm.

***Talaromyces bawangicus*** L. Wang, *sp. nov.*
MycoBank No: MB 862572
(Fig. 2)

**Etymology**. The specific epithet refers to the locale where the ex-type strain was isolated.

**Holotype**. CHINA, HAINAN: Changjiang, Bawang Ridge Nature Reserve, from soil, 19°7′53″N 109°8′2″E, 1,000 m, 30 Aug. 2019, *H.-J. Chen* CJ2-3, ex-type culture CGMCC 3.29770 (*holotype*: HMAS 354226, from dried culture of CGMCC 3.29770 on CYA). GenBank: *BenA* = PX995142, *CaM* = PX995144, ITS = PX983013, *Rpb2* = PX995146.

**Diagnosis.** This new taxon is characterized by restricted growth on CYA, YES, Cz and OA, moderate growth and sporulation on MEA, sparse sporulation on CYA, Cz, OA, while abundant sporulation on YES, apically swollen stipes, biverticillate penicilli with loosely positioned metulae, ampuliform phialides with thick collula, and ovoid, ellipsoidal to fusiform, smooth-walled conidia.

### Description

Colonies 9–10 mm diam on **CYA** at 25 °C after 7 d, low, plane, margins irregular; texture densely floccose to velvety; sporulation limited, Pea Green (R. Pl. XLVII); mycelium white mingled with a dimmed light yellow tint; no exudate; soluble pigment limited, pale yellow; reverse Green-Yellow (R. Pl. V). Colonies 21–22 mm diam on **MEA** at 25 °C after 7 d, low, plane, margins regular; texture densely floccose, overlaid with short mycelia; sporulation moderate, Light Grayish Olive (R. Pl. XLVI); mycelium white; no exudate and soluble pigment; reverse Buff Yellow (R. Pl. IV). Colonies 12–13 mm diam on **YES** at 25 °C after 7 d, slightly deep, radially plicate slightly, margins regular; texture strictly velvety; sporulation abundant, Andover Green (R. Pl. XLVII); mycelium white; exudate and soluble pigment absent; reverse Buff Yellow (R. Pl. IV). Colonies 8–10 mm diam on **Cz** at 25 °C after 7 d, low, plane, margins submerged, irregular; texture velvety; sporulation sparse, Cossack Green (R. Pl. VI); mycelium white; no exudate and soluble pigment; reverse Olive-Gray (R. Pl. LI). Colonies 12–14 mm diam on **OA** at 25 °C after 7 d, low, plane, sparse, margins fimbriate; texture velvety; sporulation sparse, Grass Green (R. Pl. VI); mycelium white; no exudate and soluble pigment; reverse pale yellow. No growth on CYA at **37 °C** and **5 °C** after 7 d.

Conidiophores born from aerial and surface hyphae; stipes (50–) 130−200 (–350) × 3−3.5 µm, smooth-walled, usually apically vesiculate up to 4 µm; penicilli biverticillate, divergent; metulae (2–) 4–6 per verticil, unequal in length, 8–12 × 3–4 µm; phialides 2–6 per verticil, ampuliform, unequal in length, 8–12 × 2.5–3 µm, with thick collula; conidia ovoid, ellipsoidal to fusiform, 3–5 × 2–3 µm, smooth-walled, born in short disordered chains.

Additional strains: CHINA, TIBET: Shigaze, Dingjie County, Chentanggou, from soil, 87°25′48″E 27°52′12″N, 2,245 m, 12 Sept. 2015, *L. Wang* NL1-12 = CGMCC 3.29771. GenBank: *BenA* = PX995143, *CaM* = PX995145, ITS = PX983014, *Rpb2* = PX995147.

## Discussion

Species in the section *Subinflati* usually grow slowly and produce white mycelium or white with a dimmed yellow tint rather than the vivid green-yellow, orange-yellow or orange mycelium as other *Talaromyces* species, and the colony texture on MEA is often floccose or densely floccose, except these, there are hardly other morphological characters in common among the species in this section. The morphological characters varied greatly among the members, thus it is easy to distinguish from one another using morphology. For example, *T. sinensis* is the only member that does not grow on CYA and YES (Peng et al., 2025). Also, *T. resedanus* grows at 37 °C, and *T. palmae* produces synnemata, whereas the other members do not grow at 37 °C and do not produce synnemata (Yilmaz et al., 2014; Sun et al., 2020). Only two members produce strictly monoverticillate penicilli in this section, namely, *T. resedanus* and *T. paecilomycetoides*, while the latter grows much more slowly than *T. resedanus* and produces a portion of single phialides directly on hyphae or on degenerated stipes, bearing much larger conidia than those of *T. resedanus* (3.0–) 6.5 × 3.6 (−9.5) × 1.5–5.0 µm *vs.* 2–3 × 1.5–2 μm) (Sun et al., 2020; Zhang et al., 2023). Although *T. parapalmae* and *T. tzapotlensis* both produce biverticillate and complex penicilli, *T. tzapotlensis* grows much faster with more abundant conidia than *T. parapalmae*, and *T. parapalmae* bears a portion of monoverticillate penicilli, whereas *T. tzapotlensis* does not (Peterson & Jurjević, 2017; Zhang et al., 2024).

The proposed new species, *T. bawangicus* and the other three members, namely, *T. guizhouensis*, *T. jiangxiensis*, *T. sinensis*, produce strictly biverticillate penicilli (Sun et al., 2020; Zhang et al., 2023; Peng et al., 2025). *Talaromyces bawangicus* can be readily distinguished from *T. guizhouensis* by its strictly velvety colony texture, and much abundant conidia on YES and apically vesiculate stipes, and from *T. sinensis* by its positive growth on CYA and YES and ampuliform phialides. The phylogenies show that *T. bawangicus* is closely related to *T. jiangxiensis*, though the colony data are lacking for comparison, the microscopic characters are obviously different between them. For instance, *T. jiangxiensis* forms typical appressed biverticillate penicilli and acerose phialides bearing spiny conidia, whereas the new species produces divergent biverticillate penicilli and ampuliform phialides bearing smooth-walled conidia, in addition, the stipes of the new species are usually apically vesiculate, whereas those of *T. jiangxiensis* are not. In the original description (Yaguchi, Miyadoh & Udagawa, 1993), *T. subinflatus* also produces apically vesiculate stipes bearing biverticillate penicilli with divergent metulae and ampuliform phialides as *T. bawangicus*, whereas the diameters of its stipe vesicles are much larger than those of the new species (6–9 µm *vs.* 4 μm) and its metulae are apically swollen as well, while those of the new species are not. Besides the biverticillate penicilli, *T. subinflatus* also produces a portion of terverticillate and monoverticillate ones, and the conidia of *T. subinflatus* are subglobose to ellipsoidal in shape and smaller (2–3.5  × 2–2.5 μm), whereas the conidia of the new species are ovoid, ellipsoidal to fusiform and larger (3–5  × 2–3 μm). In addition, *T. bawangicus* grows much faster than *T. subinflatus* (Cz: 8–10 mm *vs.* three mm; MEA: 21–22 mm *vs.* 14–15 mm). All the evidence shows the distinctiveness of the new taxon in sect. *Subinflati*.

## Conclusions

In this study, we used the traditional taxonomic method integrated with molecular phylogenetic analysis in isolating and proposing a new species in section *Subinflati*, which is the smallest section in *Talaromyces*. It is a hard work to isolate rare fungal species. The metabarcoding technique has been popular in the studies on fungal diversity, especially using ITS region, but it has a low resolution in distinguishing different species of many common fungal genera, such as *Apergillus*, *Penicillium*, *Talaromyces*, *etc.*, which may give a result inconsistent with the actual fungal diversity in nature. Thus, the traditional method in isolation and cultivation is still necessary. In addition, we have hitherto found no reports on the enzymes and secondary metabolites produced by the species in section *Subinflati*. Possibly, the whole genome sequencing and analysis might give an answer.

## Supplemental Information

10.7717/peerj.21395/supp-1 Supplemental Information 1 ML phylogram inferred from partial *BenA* sequences Bootstrap percentages over 70% derived from 1,000 replicates are indicated at the nodes. Bar = 0.05 substitutions per nucleotide position.

10.7717/peerj.21395/supp-2 Supplemental Information 2 ML phylogram inferred from partial *CaM* sequences Bootstrap percentages over 70% derived from 1,000 replicates are indicated at the nodes. Bar = 0.05 substitutions per nucleotide position.

10.7717/peerj.21395/supp-3 Supplemental Information 3 ML phylogram inferred from partial Rpb2 sequences Bootstrap percentages over 70% derived from 1,000 replicates are indicated at the nodes. Bar = 0.05 substitutions per nucleotide position.

10.7717/peerj.21395/supp-4 Supplemental Information 4 ML phylogram inferred from partial ITS sequences Bootstrap percentages over 70% derived from 1,000 replicates are indicated at the nodes. Bar = 0.02 substitutions per nucleotide position.

10.7717/peerj.21395/supp-5 Supplemental Information 5 The sequence matrices used for the phylogenetic analyses The alignments of *BenA-CaM-Rpb2-* ITS sequences, *BenA* sequences, *CaM* sequences, *Rpb2* sequences, ITS1-5.8S-ITS2 sequences. Use the software MEGA to view them.

## Additional Information and Declarations

### Data Availability

The following information was supplied regarding data availability: The data is available in the Supplemental Files.

### New Species Registration

The following information was supplied regarding the registration of a newly described species: Talaromyces bawangicus is registered at MycoBank: 862572.
