# Supplementary figures and images for: A new species of Talaromyces sect. Subinflati discovered in China
Source: PeerJ. 2026 Jun 1;14:e21395. doi: 10.7717/peerj.21395 (PMC13235687; doi:10.7717/peerj.21395)

Fig. S1. *BenA*

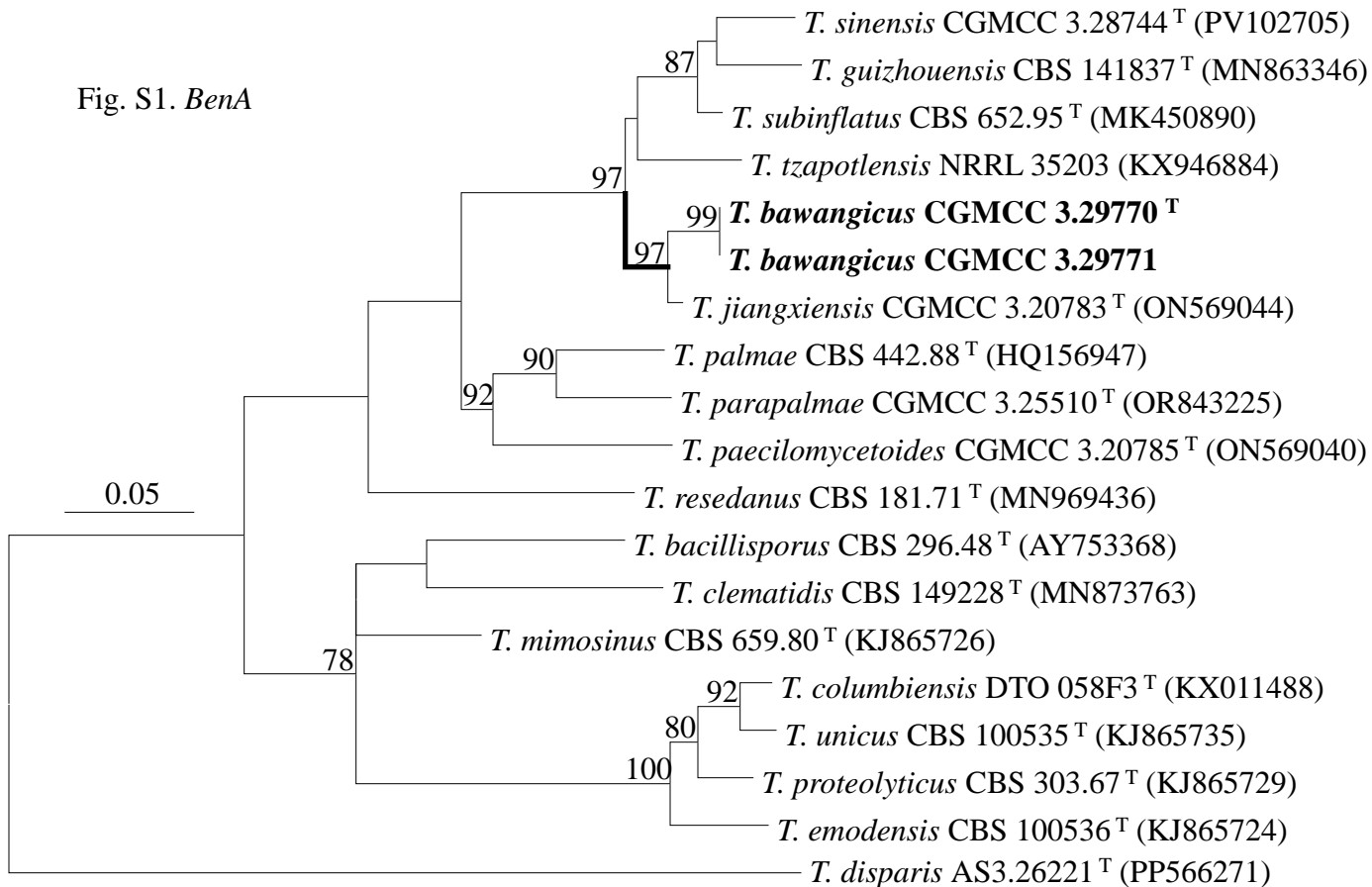

Supplement: Supplemental Information 1 — Bootstrap percentages over 70% derived from 1,000 replicates are indicated at the nodes. Bar = 0.05 substitutions per nucleotide position. [file peerj-14-21395-s001.pdf]

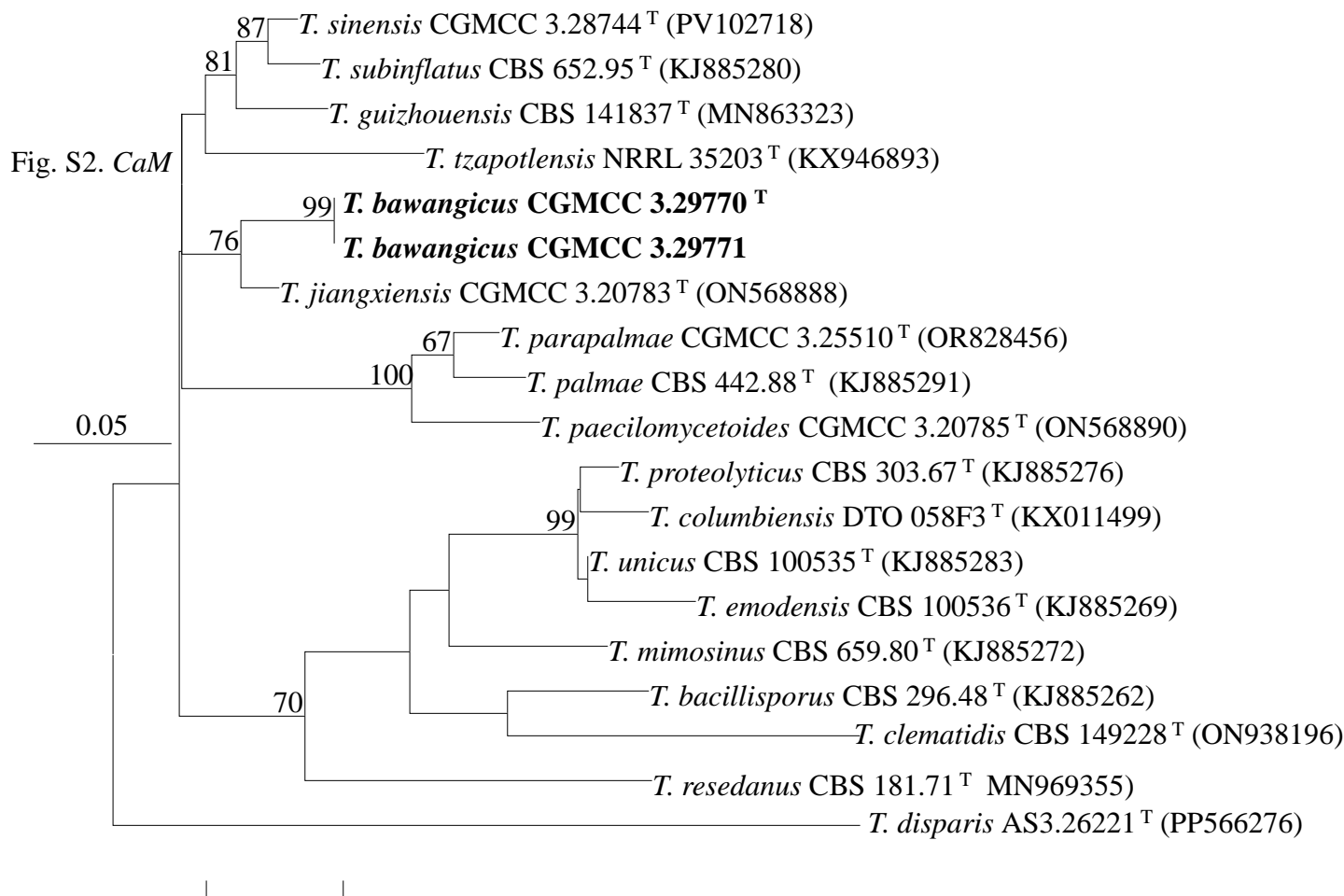

Supplement: Supplemental Information 2 — Bootstrap percentages over 70% derived from 1,000 replicates are indicated at the nodes. Bar = 0.05 substitutions per nucleotide position. [file peerj-14-21395-s002.pdf]

Fig. S3. *Rpb2*

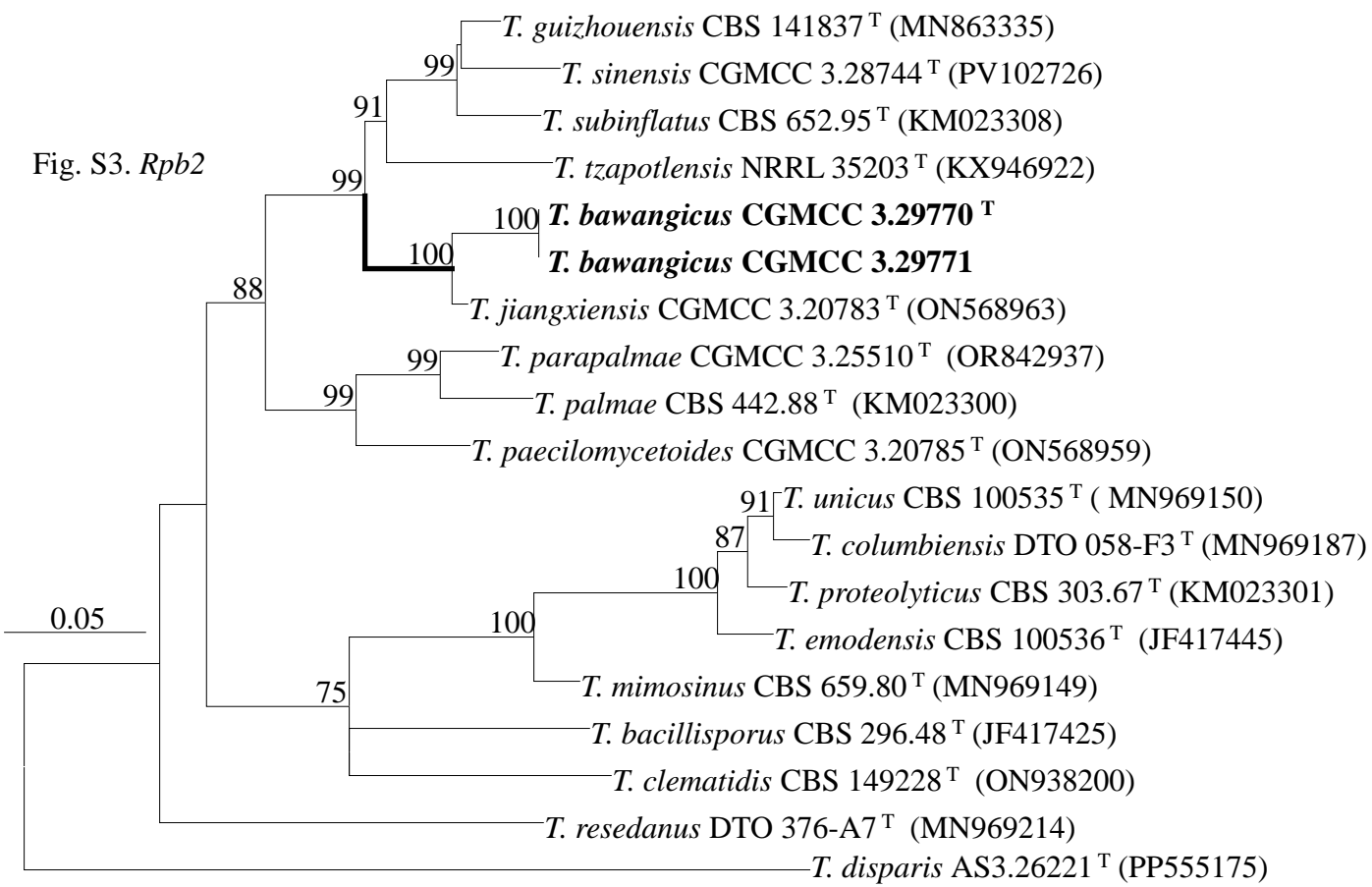

Supplement: Supplemental Information 3 — Bootstrap percentages over 70% derived from 1,000 replicates are indicated at the nodes. Bar = 0.05 substitutions per nucleotide position. [file peerj-14-21395-s003.pdf]

Fig. S4. ITS

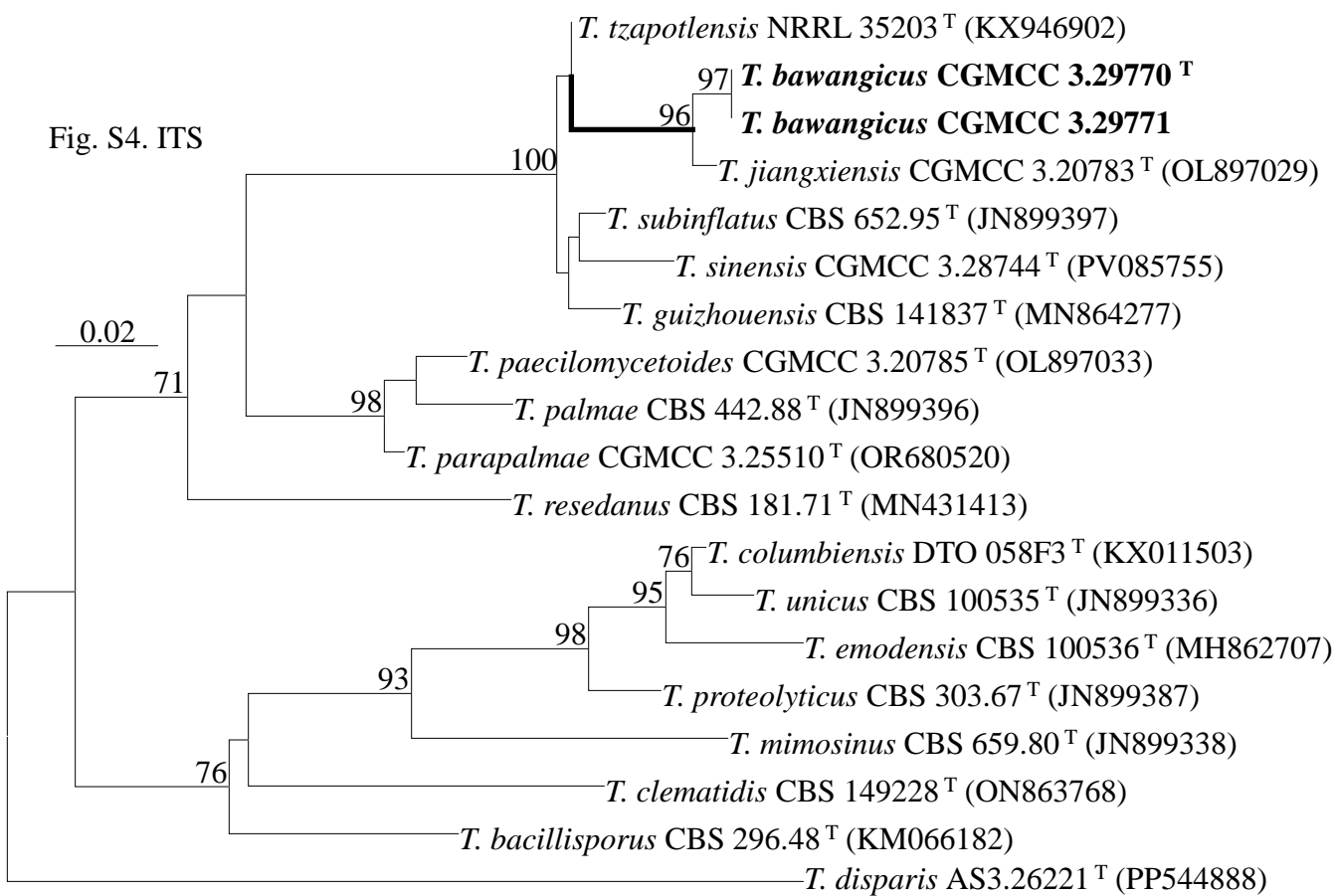

Supplement: Supplemental Information 4 — Bootstrap percentages over 70% derived from 1,000 replicates are indicated at the nodes. Bar = 0.02 substitutions per nucleotide position. [file peerj-14-21395-s004.pdf]
